# Am I (Not) Perfect? Fear of Failure Mediates the Link Between Vulnerable Narcissism and Perfectionism

**DOI:** 10.3390/bs15091214

**Published:** 2025-09-06

**Authors:** Sabrina Schneider, Sabrina Kornberger, Angela Aja Aßmuth, Andreas Mokros

**Affiliations:** Faculty of Psychology, FernUniversität in Hagen, Universitätsstr. 37, 58097 Hagen, Germanyandreas.mokros@fernuni-hagen.de (A.M.)

**Keywords:** narcissism, perfectionism, fear of failure, failure avoidance, approach-avoidance task

## Abstract

(1) Background: Perfectionism, generally conceptualized as a striving for flawlessness, can lead to maladaptive thoughts, feelings, and behavior. Both grandiose narcissism (GN) and vulnerable narcissism (VN) represent relevant personality dispositions for perfectionism. There is reason to assume that GN and VN predispose to different forms of perfectionist cognition and behavior. It remains unclear, however, whether GN and VN are indeed distinctly associated with different aspects of perfectionism and—if so—why. (2) Methods: We explored relationships between GN, VN, other-oriented, and socially prescribed perfectionism in a convenience sample of 210 adults (59% female) and further examined whether these relationships were mediated by distinct aspects of fear of failure, which has been identified as a critical driver for perfectionism. Moreover, we assessed implicit failure avoidance by means of response latencies obtained in a lexical approach-avoidance task. (3) Results: Our results indicate that perfectionist styles discriminate GN from VN whereby GN predict other-oriented and VN predict socially prescribed perfectionism. The latter relationship was largely mediated by social aspects of fear of failure (e.g., the fear of important others losing interest). In contrast, fear of failure did not explain the link between GN and other-oriented perfectionism. Furthermore, only VN was exclusively related to faster implicit failure avoidance. (4) Conclusions: This pattern of results suggests distinct mechanisms for GN and VN in the context of perfectionism. Our study provides support for the theoretical separation of GN and VN as relatively distinct phenotypes of narcissism and adds to clinical research linking GN and VN with different types of psychopathology.

## 1. Introduction

Perfectionism is a complex, multidimensional personality trait that can contribute to severe intra- and interpersonal problems. Individuals high in perfectionism are often described as rigid, radical, and overly critical in their expectations and self-evaluations (i.e., setting unrealistically high standards Frost et al., 1993). They are preoccupied with perfection and driven to achieve it (Frost et al., 1990; Smith et al., 2022). A large body of research has shown that high levels of perfectionism bear a variety of negative consequences, ranging from psychological maladjustment (e.g., Chęć et al., 2025; Collin et al., 2020; Smith et al., 2022; Stricker et al., 2022) to clinically relevant outcomes and psychopathology, including depression and anxiety (e.g., Cludius et al., 2022; Collin et al., 2020; Hewitt & Flett, 1990, 1991; Simon et al., 2025), obsessive–compulsive disorder (Cludius et al., 2022), eating disorder (e.g., Longo et al., 2024), addiction (Hewitt & Flett, 1991; Maftei & Opariuc-Dan, 2023), and different types of personality disorders (e.g., Hewitt & Flett, 1991; Redden et al., 2023). Perfectionism was even identified as a risk factor for suicidal ideation (Robinson et al., 2022; Sommerfeld & Malek, 2019). Besides these intrapersonal difficulties, previous research implies that interpersonal problems also arise from perfectionism, for instance behavior involving control, manipulation, domination, or mistrust of others (e.g., R. W. Hill et al., 1997; Stoeber, 2015, 2018; Visvalingam et al., 2024).

Various conceptualizations of perfectionism have been developed over the years, which—although based on different taxonomies—usually describe both maladaptive or “unhealthy” and adaptive or “healthy” elements of perfectionism (cf. Frost et al., 1990, 1993; Hewitt & Flett, 1991; Slade & Owens, 1998). A widely adopted concept was introduced by Hewitt and Flett (1991), who distinguished three broad forms or dimensions of perfectionism: self-oriented, other-oriented, and socially prescribed perfectionism. Self-oriented perfectionism covers self-directed behaviors such as setting high standards for oneself accompanied by strict evaluations and self-censorship of one’s own behavior (Hewitt & Flett, 1991; A. P. Hill et al., 2010). This component of perfectionism has been linked to lower levels of extraversion and higher levels of conscientiousness, self-interest, and self-monitoring (Stoeber, 2015, 2018). Self-oriented perfectionists pursue excellence in their own performance and attempt to avoid failure at all costs (Hewitt & Flett, 1991; A. P. Hill et al., 2010; Smith et al., 2022). In contrast, other-oriented perfectionism comprises beliefs and expectations about the abilities and competence of others (Hewitt & Flett, 1991). It involves similar behavior to self-oriented perfectionism, but directed at other persons: Individuals with pronounced levels of other-oriented perfectionism set overly high, unrealistic standards for others and tend to judge their performance stringently.

Other-oriented perfectionism has been related to low agreeableness, high self-regard, increased self-esteem, and little regard for others, as well as antisocial and uncaring traits (Juwono et al., 2023; Stoeber, 2015, 2018). The third dimension, socially prescribed perfectionism, arises from the need to meet standards and expectations that are presumably held by others (Hewitt & Flett, 1991; Smith et al., 2022). These individuals believe that other people hold overly demanding and uncontrollable standards of them, critically evaluate their performance, and pressure them to be perfect. Hence, they strive for perfection based on their assumptions about others’ expectations (Smith et al., 2022). Unsurprisingly, socially prescribed perfectionism is associated with a broad range of adverse clinical outcomes, including anxiety and mood disorders, somatoform disorder, and substance abuse (e.g., Hewitt & Flett, 1991; Juwono et al., 2023). Personality traits related to socially prescribed perfectionism include neuroticism and lower levels of both agreeableness and extraversion (Stoeber, 2015).

Moreover, socially prescribed perfectionism has been found to correlate with low self-esteem, negative self-regard (Moroz & Dunkley, 2015; Stoeber, 2015), and traits that are prevalent in antisocial personality disorder (e.g., hostility, callousness, deceitfulness, and impulsivity; Flett et al., 1991; Juwono et al., 2023; Stoeber, 2015). Consequently, this component has been labeled the most maladaptive or unhealthy dimension of perfectionism (cf. Juwono et al., 2023; Stoeber, 2015).

### 1.1. Perfectionism and Narcissism

Due to the maladaptive side of the construct, socially aversive personality traits are obvious candidates in the quest for dispositional traits underlying perfectionism. Consequently, researchers investigated relationships between personality traits from the dark triad (psychopathy, Machiavellianism, and narcissism; Paulhus & Williams, 2002) and the different forms of perfectionism (e.g., Hewitt & Flett, 1991; Juwono et al., 2023; Smith et al., 2016; Stoeber, 2015, 2018). Robust relationships emerged, in particular with narcissistic traits (e.g., Juwono et al., 2023; Sherry et al., 2014; Smith et al., 2016; Stoeber, 2015, 2018). In general, narcissism is characterized by exaggerated self-views, arrogance, egocentrism, dominance, and indifference towards others (e.g., Miller et al., 2021). Over the years, various models have been developed to account for the multidimensionality and heterogeneity of narcissism. One renowned conceptualization separates grandiose narcissism (GN) from vulnerable narcissism (VN; Wink, 1991). Hereby, GN is described in terms of attributes such as entitlement, aggression, high self-esteem, and dominance, whereas VN is characterized by egocentricity, need for admiration, low or contingent self-esteem, negative affectivity (shame, feelings of inadequacy, and a vigilance for failing or being criticized), and hostility toward others (e.g., Miller et al., 2011, 2021; Weiss & Miller, 2018; Wink, 1991). Both dimensions of narcissism overlap in their egocentric ideation: the disregard of and low empathy for others. Apart from this shared antagonistic core, GN and VN are substantially distinct (Miller et al., 2011, 2021; Weiss & Miller, 2018) and there is some agreement in the scientific community that they may not only represent different narcissism dimensions, but could further be considered the source of different variants or subtypes of narcissistic personality (Cain et al., 2008; Maples et al., 2025; Miller & Campbell, 2008; Miller et al., 2011).

Considering the nature of narcissistic thinking, it has been widely theorized that such individuals use perfectionism as a tool to protect and strengthen their self-esteem (e.g., cf. Morf & Rhodewalt, 2001; Smith et al., 2016). Hereby, it can be assumed that GN and VN are related to distinct perfectionist styles: grandiose narcissists place perfectionist expectations on themselves in order to satisfy their need for achievement, status, and power (e.g., cf. Miller et al., 2011, 2021; Morf & Rhodewalt, 2001; Smith et al., 2016). Furthermore, they would be expected to impose perfectionistic demands on others, accompanied by a constant dissatisfaction with the perceived flaws of other individuals, thus seeing it as their duty to rectify their imperfections (Smith et al., 2016; Stoeber, 2015). Consistent with this perspective, previous studies found GN to be positively related to self- and particularly other-oriented perfectionism (Smith et al., 2016; Stoeber, 2015; Stoeber et al., 2015). Conceptually, VN should be associated with concerns of behaving imperfectly and being judged or criticized by others—concerns central to socially prescribed perfectionism (Miller et al., 2018). Considering that the self-esteem of vulnerable narcissists depends strongly on external feedback (Miller et al., 2018; Smith et al., 2016), vulnerable narcissists would be expected to feel determined to demonstrate their perfection to others, in order to gain approval and validation (Smith et al., 2016). There is in fact some empirical support of significant associations between VN, socially prescribed perfectionism, and the non-display of imperfections (e.g., Smith et al., 2016; Sommantico et al., 2024; Stoeber et al., 2015). For example, Stoeber et al. (2015) found socially prescribed perfectionism to be positively associated with all facets of vulnerable narcissism, even after controlling for other forms of perfectionism. This pattern was later confirmed by meta-analytic evidence (Smith et al., 2016). Despite these preliminary findings, thus far it remains unclear whether the different forms of perfectionism (i.e., other-oriented and socially prescribed perfectionism) are able to discriminate GN from VN. A majority of previous studies relied exclusively on measures of GN (for exceptions see Sommantico et al., 2024; Stoeber, 2015). Moreover, extant findings are inconclusive with regard to the relationship between GN and socially prescribed perfectionism: Although some studies report a significant association (Farrell & Vaillancourt, 2019; Juwono et al., 2023), others do not (Abbasi et al., 2024; Vecchione et al., 2023). Consequently, one aim of the present study was to clarify inconsistent correlation patterns between the different perfectionism styles and GN vs. VN and to determine whether these two expressions of narcissism show different strengths of association with different aspects of perfectionism, thereby clarifying the distinct nomological networks of GN and VN (Miller et al., 2011, 2017).

### 1.2. Perfectionism and Fear of Failure

In addition to narcissistic personality traits, previous research identified another critical predisposition that drives perfectionist cognition and behavior: Beyond narcissistic traits, another key individual difference closely linked to perfectionism is *fear of failure*. This refers to the tendency to experience anxiety in situations with a potential for failure (Conroy et al., 2007), rooted in the underlying fear of not being able to achieve personally relevant goals or to display competence in performance contexts (Henschel & Iffland, 2021). According to Conroy (2001; see also Conroy et al., 2002, 2007), fear of failure comprises five components that represent different putative consequences of failure that individuals may be afraid of: (1) the fear of experiencing shame and embarrassment (FSE), (2) the fear of devaluing one’s self-estimate (FDSE; i.e., concerns about information/feedback that might challenge or lower a person’s existing self-view), (3) the fear of having an uncertain future (FUF), (4) the fear of important others losing interest (FIOLI), and (5) the fear of upsetting important others (FUIO).

Conroy et al. (2007) describe fear of failure as a “primary motivation underlying perfectionism” (p. 238). Extant empirical research largely corroborates this assertion, whereby a majority focused on the athletic or procrastination context (e.g., A. P. Hill et al., 2010; Onwuegbuzie, 2000; Sagar et al., 2010). Some findings are, however, inconsistent or even contradictory (cf., e.g., Conroy et al., 2007; A. P. Hill et al., 2010; Yosopov et al., 2024). This suggests that the relationship between perfectionism and fear of failure (and the pattern of interrelations of their respective components) is likely complex. Even though some studies indicate that all perfectionism dimensions are related to fear of failure (Sagar & Stoeber, 2009; Yosopov et al., 2024), others revealed distinct patterns of associations between the different forms of perfectionism, fear of failure in general, and its five components (Conroy et al., 2007; Hewitt et al., 2003; A. P. Hill et al., 2010; Lee et al., 2024; Onwuegbuzie, 2000). Despite some differences, these publications reported one common finding: across studies, fear of failure was consistently associated with socially prescribed perfectionism, the most maladaptive perfectionist style.

### 1.3. The Present Study

While extant research emphasizes that narcissism represents a key personality disposition for perfectionistic cognition and behavior, it remains unclear whether GN and VN exhibit clearly distinct relationships with different forms of perfectionism. Therefore, the first aim of the present study was to further elucidate the narcissism–perfectionism link, in particular to examine whether different perfectionist styles can discriminate between GN and VN. Based on narcissism theory and preliminary empirical evidence, we assumed that VN would be especially associated with socially prescribed perfectionism, whereas GN was anticipated to relate primarily to other-oriented perfectionism. In contrast, self-oriented perfectionism appears to be present in both GN and VN (Flett et al., 2014; Smith et al., 2016). Therefore, we focused on other-oriented and socially prescribed perfectionism in our study, as these dimensions were expected to provide the clearest distinction between GN and VN. The second aim of the present study was to replicate previous research addressing precedents of perfectionism, which suggests that socially prescribed perfectionism is strongly motivated by fear of failure. Theoretical accounts of perfectionism conceptualize fear of failure as a critical motivational force driving perfectionistic behavior (Conroy et al., 2007). Within narcissism theory, fear of failure has been proposed as an outcome of fragile self-regulation (Elliot & Thrash, 2001; Morf & Rhodewalt, 2001). Likewise, clinical theories of hypersensitive narcissism highlight fear of evaluation as a consequence of vulnerable narcissistic dynamics (Pincus & Lukowitsky, 2010). Taken together, fear of failure appears to serve as a bridge between dispositional narcissistic traits and perfectionism styles. Consistent with these theoretical assumptions, narcissism—particularly the vulnerable dimension—has been linked to both fear of failure and negative evaluation (Besser & Priel, 2010; Dumitrescu & De Caluwé, 2024), as well as compensatory perfectionistic cognition and behavior (Smith et al., 2016; Stoeber, 2015). Accordingly, we hypothesized that the relationship between VN and socially prescribed narcissism is mediated by social aspects of fear of failure (Hypothesis 1), which can be expected to be especially relevant to vulnerable narcissists, as they involve a feared negative evaluation by others (FIOLI, FUIO) or feelings of inadequacy (FSE). In contrast, because grandiose narcissists are less sensitive to these concerns, FIOLI, FUIO, or FSE are unlikely to explain the relationship of GN and other-oriented perfectionism. Instead, we expected FDSE to mediate this link (Hypothesis 2), as FDSE was the only fear-of-failure component predicting other-oriented perfectionism in a multiple regression analysis in a previous study (Conroy et al., 2007).

Perfectionism functions as a (potentially maladaptive) tool to avoid feared or anticipated failure (Conroy et al., 2007). Hence, highly perfectionist individuals can be expected to display the tendency to avoid failure and failure-related cues as much and as often as possible. To the best of our knowledge, none of the previous studies on perfectionism and/or fear of failure assessed behavioral measures of fear of failure, such as failure avoidance. To address this gap, we included a behavioral index of failure avoidance to examine whether GN and VN exhibit distinguishable patterns not only in self-reported perfectionism and fear of failure, but also in observed avoidance behavior. In psychological research, approach and avoidance tendencies are usually investigated by means of so-called approach-avoidance tasks (AATs; for an overview, see Krieglmeyer & Deutsch, 2010). In this paradigm, participants are asked to either approach (i.e., move toward) or avoid (i.e., move away from) certain valence-laden target stimuli presented to them. Depending on the AAT variant, these movements are executed by the entire body, by body parts (e.g., by pulling or pushing a joystick), or by pressing designated approach or avoidance keys on a keyboard. The basic idea of AATs is that approach responses occur faster when the stimulus presented has a positive valence for the respondent, whereas avoidance or withdrawal responses are faster when the stimulus valence is negative. Accordingly, response times in AATs are commonly interpreted as indicators of a stimulus’s implicit valence (Krieglmeyer & Deutsch, 2010) or its interpretation (Phaf et al., 2014), as well as the individual’s underlying preference to approach or avoid it. Based on the assumption that failure is particularly aversive for vulnerable narcissists, we expected to find vulnerable narcissism linked with an accelerated avoidance of failure cues presented in an AAT (Hypothesis 3). The hypotheses were preregistered before data collection commenced (https://aspredicted.org/6kq7p.pdf, accessed on 1 September 2025)^1^.

## 2. Methods

### 2.1. Sample

Participants were recruited between January and April 2019 from the host university and external sources, including social networks such as Facebook and WhatsApp, with a total of 330 individuals initially enrolling in the study. Inclusion criteria required participants to be at least 18 years old, possess fluent language proficiency, and have the physical and mental ability to participate in an online study lasting approximately 30 min. Completion of the AAT paradigm also required a desktop PC or notebook with a keyboard including a numeric keypad. A total of 108 participants were excluded due to incomplete experimental participation (e.g., technical issues such as not using the required keyboard) or indications of invalid responses (i.e., unrealistically fast or slow reaction times: see Data Preprocessing and Statistical Analyses section). Additional exclusions were made for participants who reported inattentive or dishonest responding after the study (*n* = 8) or displayed invariant responses on self-report scales used to derive dependent and independent variables (*SD* = 0; *n* = 1).

The final sample consisted of 213 participants (*n* = 126 female), with a mean age of 32 (*SD* = 11, *range* = 18–67) years. A majority of participants (*n* = 92, 43%) stated a high school diploma (German Abitur) as their highest degree, 19% (*n* = 39) reported holding a master’s degree equivalent, and a further 17% (*n* = 36) reported holding a bachelor’s degree. In sum, 128 (60%) were married or in a relationship at the time of participation. About 40% (*n* = 87) of the sample were full-time employees. A further 33% (*n* = 69) of the participants reported being university students at the time of participation.

### 2.2. Design and Procedure

Data were collected online. The study protocol was approved by the host university’s local institutional review board. Participants were informed about ethical and data security concerns (e.g., anonymity and the possibility to discontinue from study participation at any time), the study procedure, duration, and technical requirements in the beginning. To ensure reliable response-time measurements, completion of the experimental part (AAT) required a computer keyboard (desktop or laptop) with a numeric keypad and the free Inquisit web player (version 5, Millisecond Software, LLC, Seattle, WA, USA). Participants were also instructed to complete the study in a quiet environment with a stable internet connection, alone, without external distractions (e.g., background music or radio), and to close all unrelated web applications. After providing informed consent, participants first completed the self-report scales (see below), followed by the experimental procedure (i.e., the AAT). Stimulus presentation and response recording were controlled by Inquisit (Milliseconds Software, LLC). After completing the AAT, participants rated each of the 24 target words used in the AAT with respect to their valence and were than thanked and debriefed.

### 2.3. Materials and Measures

#### 2.3.1. Grandiose and Vulnerable Narcissism

We assessed grandiose (GN) and vulnerable narcissism (VN) with the Pathological Narcissism Inventory (PNI; Pincus et al., 2009), a self-report scale consisting of 52 items that are answered on a 6-point Likert scale (1 = does not apply to me at all, 2 = does almost not apply to me, 3 = does rather not apply to me, 4 = rather true for me, 5 = quite true for me, 6 = completely true for me). A German version comprising 54 items was constructed and validated by Morf et al. (2017). Items are grouped into seven subscales, which according to validation studies (Morf et al., 2017; Pincus et al., 2009) capture either GN or VN: contingent self-esteem (VN), exploitativeness (GN), self-sacrificing self-enhancement (GN), hiding the self (VN), grandiose fantasy (GN), devaluing (GN), and entitlement rage (GN). Morf et al. (2017) reported good-to-excellent reliability indices for the PNI subscales (*α* = 0.84–0.92) and the PNI total score (*α* = 0.94). High inter-factor correlations for GN and VN were also reported (Wright et al., 2010).

#### 2.3.2. Perfectionism

We used the German version (Altstötter-Gleich, 2018) of the Multidimensional Perfectionism Scale (MPS; Hewitt et al., 1991). The MPS comprises 45 items, which can be divided into the abovementioned dimensions of self-oriented perfectionism (SOP; *α* = 0.89), other-oriented perfectionism (OOP; *α* = 0.79), and socially prescribed perfectionism (SPP; *α* = 0.86), which are measured by 15 items each. Items are rated on a 6-point Likert scale (1= strongly disagree, 2 = disagree, 3 = somewhat disagree, 4 = somewhat agree, 5 = agree, 6 = fully agree). Eighteen inverted items were recoded before computing the scale scores. Temporal stability (*r_tt_* = 0.75–0.88) and internal consistencies in the three subscales (see above) were reported to be good (Hewitt et al., 1991).

#### 2.3.3. Fear of Failure

We implemented a German adaptation of the Performance Failure Appraisal Inventory (PFAI; Conroy et al., 2002) in order to assess different aspects of fear of failure. The PFAI consists of 25 items that capture fear with regard to five different aversive consequences of failure: fear of experiencing shame and embarrassment (FSE), fear of devaluing one’s self-estimate (FDSE), fear of having an uncertain future (FUF), fear of important others losing interest (FIOLI), and fear of upsetting important others (FUIO). Items of the PFAI are answered on a 5-point Likert scale (1 = do not believe at all to 5 = believe 100% of the time). As no German translation of the PFAI was available by the time this study was conducted, we prepared a German version of the 25 items by means of the forward–backward translation method with four independent translators (including one native speaker of the English language). Conroy et al. (2007) reported internal consistency estimates for the PFAI subscales ranging from *α* = 0.73 (FDSE) to *α* = 0.83 (FIOLI). For the sake of conciseness, we report only results for the PFAI dimensions FDSE, FIOLI, FUIO, and FSE that were relevant for testing our study hypotheses.

#### 2.3.4. Failure Avoidance

**Figure 1:**
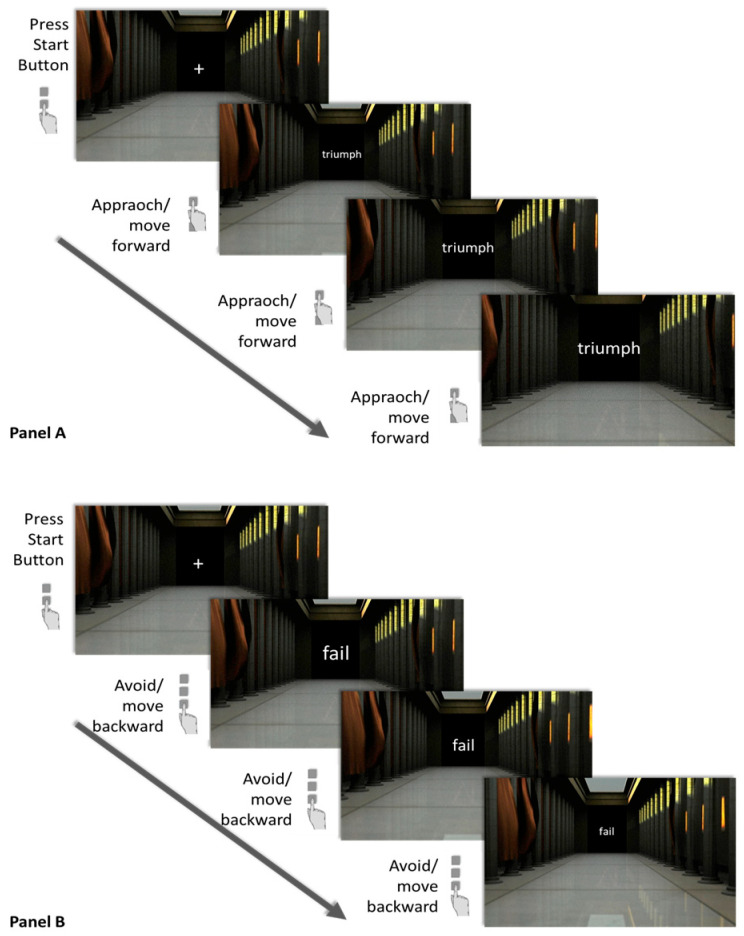
Illustration of the approach–avoidance task design used in the present study. Note: The stimulus–response-compatible task condition is shown. The hand symbol shown below each screen indicates the correct response (button press) in the respective task condition. (**A**)—exemplary trial sequence for approaching success stimuli; (**B**)—exemplary trial sequence for avoiding failure stimuli.

We assessed failure avoidance with a lexical version of the visual approach–avoidance of the self task (VAAST; Rougier et al., 2018), which has been reported to be suitable for online applications (Aubé et al., 2019). The VAAST used in the present study portrays stimuli of different categories, here 24 words indicating either success (e.g., “triumph,” “acknowledgment”) or failure (e.g., “defeat,” “disgrace”), within a three-dimensional environment on screen. Participants are instructed to press designated response buttons to virtually approach the stimuli of one category while virtually avoiding the other. Hence, the respective target response depends on the respective experimental block. When the “approach” key is used, the word stimulus and its surrounding 3D environment are gradually enlarged, creating the impression of the participant moving toward the stimulus (see panel A of Figure 1). When the “avoidance” key is pressed, word stimulus and environment in turn become gradually smaller, creating the visual impression of the person moving backward and away from the stimulus (see panel A of Figure 1). Like most AATs, the VAAST consists of two task blocks: in the stimulus–response (SR)-compatible block (depicted in Figure 1), subjects are instructed to avoid (i.e., press the “avoid” key and move away from) negative stimuli (i.e., failure words) and approach (use the “approach” key in order to move toward) positive stimuli (i.e., success words), whereas they are required to execute the exact opposite response (i.e., approach failure words and avoid success words) within the SR-incompatible block. In order to initiate a new trial in which a new word stimulus is presented on screen, participants press a start button on the keyboard and the stimulus then appears within a jittered time interval of 600 to 1800 ms. Participants were instructed to respond as quickly as possible after stimulus onset.

Given the two stimulus categories used in the present study, the VAAST included the following four conditions, with two SR-compatible conditions—(1) approach–success and (2) avoid–failure, and two SR-incompatible conditions: (3) approach–failure and (4) avoid–success. Our analyses focus on the avoid–failure condition, which represents the critical scenario in which active avoidance (i.e., a withdrawal response) occurs. Most studies implementing AAT paradigms compute difference scores (e.g., the latency difference between approach and avoidance responses for the same stimulus type), which are commonly interpreted as measures of stimulus valence (Krieglmeyer & Deutsch, 2010; Phaf et al., 2014). In the present study, however, we were specifically interested in behavioral correlates of fear of failure, namely active avoidance of failure stimuli, rather than the overall implicit valence of these stimuli. Active avoidance can be observed directly within the SR-compatible block, where faster withdrawal (shorter reaction times) from failure words indicates a tendency toward or facilitation of active failure avoidance. To conceptually differentiate between different aspects of avoidance, following suggestions by Najmi et al. (2010), we analyzed AAT response times separately for each task condition and examined their associations with the traits of interest (e.g., VN, GN). This approach is more appropriate for studies testing specific hypotheses regarding particular types of behavior (e.g., active avoidance of fear-related stimuli), rather than using the AAT as a general implicit measure (e.g., Najmi et al., 2010; Struijs et al., 2017).

Twelve words (seven nouns, two adjectives, and three verbs) per stimulus category were included in the AAT, whereby each word was presented twice per task block, resulting in 48 SR-compatible and 48 SR-incompatible trials. Six additional trials per block with words that were not included in the main task blocks were used as practice trials. Participants could take a self-timed break between task blocks. The complete list of word stimuli and the experimental code can be retrieved from https://osf.io/w2459/ (accessed on 1 September 2025). The word stimuli were selected from a pool of 96 words of the two semantic categories success and failure, which were rated with respect to their valence (from very unpleasant to very pleasant) on 7-point response scales by 27 adult raters who did not participate in the main study. Based on these ratings, 12 final words per category (i.e., success and failure, respectively) were chosen that discriminated best between the two stimulus categories with respect to their valence (success words: *M* = 5.88, *SD* = 0.26; failure words: *M* = 1.81, *SD* = 0.30). The final 24 success and failure words on average did not differ with regard to their length (represented by the number of letters: *t*(22) = −0.847, *p* = 0.41) and their word frequency in the German language: *t*(22) = 1.164, *p* = 0.26.^2^ Thus, distinct responses to word stimuli denoting success or failure could be ascribed to differences in their implicit valence rather than to other stimulus features (such as word length or frequency) that are unrelated to their semantic meaning.

**Stimulus Ratings.** Upon completing the AAT, participants were once again presented with the 24 words. They were asked to rate them with respect to their subjective valence on a 7-point Likert-type scale from 1 (very negative) to 7 (very positive). We included these valence ratings to conduct AAT manipulation checks (see Results).

### 2.4. Data Preprocessing and Statistical Analyses

In line with joystick-based AAT studies (Rinck & Becker, 2007), we calculated median RTs for all trials belonging to the same VAAST condition. Only latencies of correct responses were analyzed. As RTs obtained in AAT experiments are usually positively skewed, we applied an outlier cut-off to normalize the RT data distribution and to exclude overly fast and overly slow responses in the VAAST from further analyses (below 200 ms/above 5000 ms; these cut-offs are based on Klein et al., 2011, and also accord with recent publications that include the VAAST, e.g., Schneider et al., 2025). Participants were further excluded if reaction patterns showed signs of distraction (i.e., more than 30% incorrect responses per task block).

As a first analytic step, we conducted tests of descriptive statistics and zero-order correlations among all study variables (see Table 1). Bivariate relationships between narcissistic traits (GN, VN), MPS scores (socially prescribed, other- and self-oriented perfectionism), and the hypothesis-relevant scale scores of the PFAI provide a first impression about relationships between the different aspects of narcissism, perfectionism, and fear of failure. Exploratory follow-up multiple regression analyses of socially prescribed and other-oriented perfectionism were carried out in order to examine the incremental predictive value of GN and VN for both forms of perfectionism.

We then estimated mediation models and conducted a simple linear regression analysis to test our preregistered hypotheses. First, we tested the assumptions that certain aspects of fear of failure represent a connecting link between VN and socially prescribed perfectionism (H1). To this end, the fear of important others losing interest (FIOLI), the fear of upsetting important others (FUIO), and the fear of shame and embarrassment (FSE) served as mediators, VN as a predictor, and socially prescribed perfectionism as the outcome variable. Because the different fears of failure can be present at the same time, FIOLI, FSE, and FUIO were included as parallel mediators in the model. We then tested the hypothesis (H2) that FDSE mediates the link between GN (predictor) and other-oriented perfectionism (outcome). Finally, we hypothesized that vulnerable narcissism predicts avoidance-of-failure stimuli in the AAT (H3). To probe this assumption, we tested a simple regression model with response latencies on failure words in the SR-compatible AAT condition (which represent failure avoidance) as outcome variable, whereby VN was included as a predictor variable. Although our hypotheses involved directional predictions, we applied two-sided significance tests as default.^3^ Significance levels of α = 0.05 (for the mediation/regression models) and *α* = 0.01 (for zero-order correlations) were applied. Data and analysis scripts can be retrieved from https://osf.io/w2459/ (accessed on 1 September 2025).

### 2.5. Power Considerations

The preregistered sample size of *N* = 132 represents the minimum number of cases to detect moderate bivariate correlations (*r* ≥ 0.30) with power of 0.80 and *α* = 0.01, and was calculated using G*Power version 3.1 (Faul et al., 2009). As we intended further to conduct regression-based mediation analyses, we used the simulation-based Shiny app provided by Schoemann et al. (2017). The a-priori power analysis for a mediation model with three parallel mediators (cf., H1) indicated a minimum sample size of *N* = 250 to detect an indirect effect of FIOLI, a minimum sample size of *N* = 190 to detect an indirect effect of FUIO, and a minimum sample size of *N* = 210 to detect an indirect effect of FSE with a power of ≥0.80 (*α* = 0.05).^4^ The a-priori power analysis for a mediation model with one variable mediating the link between GN and other-oriented perfectionism (FDSE, see H2) indicated a minimum sample size of *N* = 180 in order to detect an indirect effect of FDSE with a power of at least 0.80 (*α* = 0.05).

## 3. Results

### 3.1. Distinction of GN and VN Based on Their Relationships to Perfectionism

Table 1 displays descriptive statistics and bivariate interrelations between the core study variables.

In line with previous research, both GN and VN showed relationships with self-oriented, other-oriented, and socially prescribed perfectionism. Notably, other-oriented perfectionism was related more closely to GN compared to VN (∆*r* = 0.09, *z* = 1.67, *p* = 0.044). Socially prescribed perfectionism, in contrast, was tied closely to VN, but much less so to GN (∆*r* = 0.24, *z* = 4.89, *p* < 0.001). These discrepancies between GN and VN in their relationships with perfectionistic styles were not observed for self-oriented perfectionism, which displayed mediocre correlations with both GN (*r* = 0.36) and VN (*r* = 0.34; ∆*r* = 0.02, *z* = 0.39, *p* = 0.35, respectively).

As previous research has shown that the PNI, including its GN subscale, is strongly oriented towards narcissistic vulnerability (e.g., Miller et al., 2017), we performed control analyses to assess how each narcissism dimension relates to perfectionism when the influence of the other dimension is excluded. To this end, we conducted three multiple regression analyses with GN and VN as simultaneous predictors in order to ascertain their incremental predictive value for the three types of perfectionism. The results of these analyses, displayed in Table 2, showed that other-oriented and socially prescribed perfectionism discriminated well between GN and VN, with GN (but not VN) predicting other-oriented perfectionism and with VN (but not GN) predicting socially prescribed perfectionism. Self-oriented perfectionism in turn was associated with both GN and VN to a similar degree.

As mentioned above—and according to expectations—higher expressions of VN were particularly associated with higher levels of socially prescribed perfectionism (*r* = 0.55). In line with previous research (Conroy et al., 2007), FIOLI, FUIO, and FSE showed considerable relationships with socially prescribed perfectionism (*r* = 0.54–0.68), but were weakly associated with self- or other-oriented perfectionism (*r* = 0.15–0.22).

**Figure 2:**
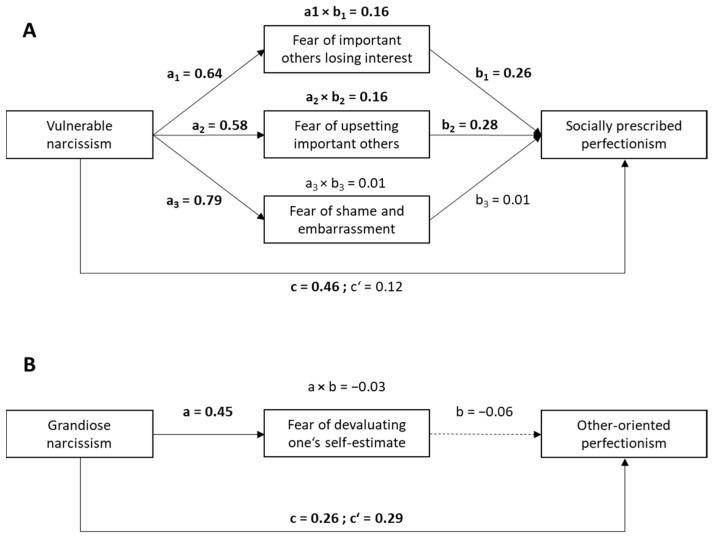
Mediation models for socially prescribed and other-oriented perfectionism. Notes: (**A**)—Direct and indirect effects of vulnerable narcissism on socially prescribed perfectionism; (**B**)—Direct and indirect effects of grandiose narcissism on other-oriented perfectionism. *N* = 213. Unstandardized path/regression coefficients are shown. c = total effect, c’ = direct effect. Bold coefficients and solid arrows indicate statistical significance (*p* < 0.01), dashed arrows and coefficients not highlighted indicate non-significant associations (*p* > 0.05), both two-sided.

The mediation analysis indicated direct and indirect effects of VN on socially prescribed perfectionism (see Figure 2A), whereby two aspects of fear of failure, FIOLI and FUIO, mediated the relationship between VN and perfectionism. The direct effect of VN on perfectionism was not significant (*b* = 0.12, 95% CI [−0.02, 0.27], *SE* = 0.06, *t* = 2.4, *p* = 0.10 two-sided), and was considerably smaller than the total effect of VN (*b* = 0.46, 95% CI [0.34, 0.57], *SE* = 0.06, *t* = 7.73, *p* < 0.001 two-sided), suggesting mediation. Notably, while FSE was considerably associated with VN, it did not function as an additional significant mediator in the model. The mediation model depicted in Figure 2A explained 54% of variance in socially prescribed perfectionism.

### 3.2. Grandiose Narcissism, Perfectionism, and Fear of Failure

Consistent with our assumptions, GN was correlated positively with other-oriented perfectionism (*r* = 0.31, *p* < 0.001). Among other aspects of fear of failure, GN was linked to FDSE (*r* = 0.35, *p* < 0.001). Our mediation analysis (Figure 2B) showed significant total (*b* = 0.26, 95% CI [0.15, 0.36], *SE* = 0.05, *t* = 4.64, *p* < 0.001 two-sided) and direct effects (*b* = 0.29, 95% CI [0.18, 0.40], *SE* = 0.06, *t* = 5.07, *p* < 0.001 two-sided) of GN on other-oriented perfectionism. There was no indirect effect (*a* × *b* = −0.03, 95% CI [−0.08, 0.07]), as FDSE was not significantly related to other-oriented perfectionism at the level of zero-order correlations (see Table 1) or within the mediation model (*b* = −0.08, 95% CI [−0.18, 0.01], *SE* = 0.05, *t* = −1.81, *p* < 0.07) (see also Figure 2B). The mediation model depicted in Figure 2B explained 11% of variance in other-oriented perfectionism, which was almost the same for the total effects model of GN, which did not include the mediation (*R*^2^ = 0.10).

Given the positive associations between other-oriented perfectionism and other aspects of fear of failure, in particular FIOLI and FUIO (see Table 1), we conducted additional exploratory mediation analyses in order to investigate whether one of these aspects—rather than FDSE—might connect GN and other-oriented perfectionism. That was not the case, as both analyses did not reveal any indirect effect (FUIO: *a* × *b* = 0.01, 95% CI [−0.03, 0.06]; FIOLI: *a* × *b* = 0.03, 95% CI [−0.02, 0.08]).

### 3.3. Narcissism, Fear of Failure, and Implicit Failure Avoidance

Before testing our third hypothesis, we conducted tests to check the plausibility of our AAT data. First, we assessed whether study participants clearly discriminated the AAT word stimuli with respect to their (un)pleasantness. A paired *t*-test of participants’ mean valence ratings of the success and failure words revealed a clear difference in subjective stimulus valence: *t*(209) = 69.56, *p* < 0.001, *d* = 4.60. Success stimuli were rated as far more positive (*M* = 6.33, *SD* = 0.50) than the failure words (*M* = 1.71, *SD* = 0.54). Second, we examined the effectiveness of our experimental manipulation in the AAT paradigm. In other words, we assessed whether the AAT produced plausible response data, with longer reaction times for stimulus-incompatible responses (i.e., approach failure words, avoid success words) compared to stimulus-compatible responses (i.e., avoid failure words, approach success words). The 2 × 2 repeated measure analysis of variance indicated a large main effect of compatibility, *F* (1, 194) = 139.83, *p* < 0.001, partial *η*^2^ = 0.42, with overall faster responses in the SR-compatible condition (*M* = 714, *SD* = 8.80 ms) compared to the SR-incompatible condition (*M* = 796, *SD* = 10.62 ms) (see also Supplemental Figure S1). This finding replicates previously reported AAT effects when the VAAST was used (Aubé et al., 2019; Rougier et al., 2018; Schneider et al., 2025) and attests to the internal validity of the experimental procedure. In addition, a main effect of word category emerged, *F* (1, 194) = 55.43, *p* < 0.001, partial *η*^2^ = 0.42, indicating that on average participants responded faster to success (*M* = 742, *SD* = 8.90 ms) compared to failure words (*M* = 767, *SD* = 9.66 ms). A significant interaction effect (*F* (1, 194) = 50.07, *p* < 0.001, partial *η*^2^ = 0.21) suggested that—while a clear SR-compatibility effect was observed in both success and failure stimuli—this effect was somewhat more pronounced for the success stimuli (see also Supplemental Figure S1 for details).

After confirming that the AAT paradigm produced the intended response bias (i.e., SR-compatibility effect), we examined the association between response latencies to failure words in the SR-compatible condition (i.e., latencies for avoiding failure words) and both grandiose and vulnerable narcissism. The pattern of zero-order correlations (see Table 1) suggested that GN was associated with generally faster responses across all AAT conditions. VN, in contrast, was associated *only* with faster responses to failure words in the SR-compatible condition (i.e., faster active avoidance of failure words). The simple regression model (*R*^2^ = 0.022, *F* (1, 207) = 4.65, *p* = 0.03, two-sided) confirmed that higher expressions of VN predicted faster avoidance of failure words (*b* = −22.96, *SE* = 10.65, *β* = −0.15, *t*(207) = −2.16, *p* = 0.03 two-sided). Notably, correlation coefficients further indicated faster success approach and faster failure avoidance with increasing levels of FSE (*r* = −0.17/−0.18), a type of fear particularly linked to VN (see Table 1).

## 4. Discussion

Perfectionism is a relevant construct for advancing the understanding of narcissistic dynamics (e.g., Casale et al., 2024; Sherry et al., 2014; Sommantico et al., 2024; for an overview, see Smith et al., 2016). More specifically, close scrutiny of perfectionism and its components may help explain differences in the clinical manifestation of grandiose and vulnerable narcissism. To that end, the core aim of the present study was to elucidate the interplay of narcissism, perfectionism, and their potential common mechanism: fear of failure. Consistent with prior research (Smith et al., 2016; Stoeber et al., 2015), our findings indicate that distinct forms of perfectionism—specifically, other-oriented and socially prescribed perfectionism—discriminate clearly between two major dimensions of narcissism (grandiose and vulnerable narcissism). More precisely, other-oriented perfectionism (demanding high standards of other people) seems to be rooted in narcissistic grandiosity, whereas socially prescribed perfectionism (believing that others set high standards and feeling the urge to live up to them) is linked to narcissistic vulnerability. Differences in these associations were statistically significant and provide further evidence for the distinct nomological networks of GN and VN (Miller et al., 2011, 2014), with the former being related to high external demands on others and the latter being linked to unrealistic internal self-demands. Although both GN and VN are linked to maladaptive outcomes, vulnerable narcissism (VN) appears to be more directly relevant to psychopathology (e.g., Pincus & Lukowitsky, 2010). This aligns with previous work framing socially prescribed perfectionism as particularly maladaptive (e.g., Stoeber, 2015).

These findings raise the question of *how* exactly GN and VN foster distinct perfectionistic styles. Based on theory (e.g., Conroy et al., 2007; Morf & Rhodewalt, 2001), we assumed that fear of failure might play a critical role in this context and examined the distinct relationship patterns between different aspects of fear of failure—namely FIOLI, FUIO, FSE, and FDSE—both narcissistic traits, and both forms of perfectionism. Consistent with our expectations, two aspects of fear of failure—FIOLI and FUIO—mediated the link between VN and socially prescribed perfectionism. Vulnerable narcissists seem to be particularly concerned with anticipated social consequences of failure, such as upsetting important others and/or losing their attention due to their own flaws and imperfections. These concerns are intertwined with perceived social pressure and expectations to be perfect, possibly driving individuals with high expressions of VN to present themselves as flawless in front of others. Interestingly, although vulnerable narcissists also seem to fear negative experiences such as shame and embarrassment (*r_VN,FSE_* = 0.70), this particular fear did not contribute to their perfectionist efforts in the present study. Instead, the potential loss of other persons’ interest or benevolence seemed to be the most critical agent in this context, whereby vulnerable narcissists might display perfectionist behavior in order to cope with these fears. While this interpretation seems plausible from a theoretical perspective, the cross-sectional design of the present study does not allow for causal conclusions to be drawn. Longitudinal studies and/or experimental manipulations of narcissistic states could help clarify the process underlying the narcissism–perfectionism connection.

With respect to GN, an entirely different picture emerged, supporting the notion that GN and VN represent distinguishable narcissistic personality variants with differential individual and social consequences (e.g., Cain et al., 2008; Miller & Campbell, 2008; Miller et al., 2011, 2021; Weiss & Miller, 2018). In contrast to VN, GN exclusively predicted other-oriented perfectionism, reflecting high standards and expectations for perfection imposed on other people (Hewitt & Flett, 1991). Mediation analyses suggested that this relationship could not be explained by any aspect of fear of failure. Although failure-related fears seem to play a role in GN (cf. Table 1), these concerns do not seem to account for the high standards grandiose narcissists hold others to. We suggest that fear of failure, at least those aspects captured by the PFAI (Conroy et al., 2002), represent mostly self-handicapping concerns that tend to evoke affective and behavioral responses that are directed inward (e.g., internalizing symptoms such as anxiety, depression, self-criticism, etc.). It seems likely that mechanisms other than fear of failure link GN to (other-oriented) perfectionism, which might include, for example, the sense of entitlement. In light of their inflated self-view, grandiose narcissists might feel entitled to the best performance of people they are involved with and expect them to live up to high standards in order to qualify for their companionship. Setting high standards for others might also represent a strategy to consolidate a superior social status, which has been suggested as an important motive in GN (Grapsas et al., 2020). On another note, antagonistic components inherent in GN might also play a role linking GN with other-oriented perfectionism: setting unrealistic standards that are virtually impossible to meet by others might legitimize narcissistic individuals to devalue them. Future research could address these and other ideas and continue the quest for factors that underlie perfectionist cognition and behavior in GN.

In addition to self-reported fears of failure and perfectionist tendencies, we assessed negative responses to failure at the behavioral level. Hereby, we measured active failure avoidance as a behavioral correlate of fear of failure by means of response latencies in an AAT. In this paradigm, we observed overall faster avoidance of failure cues and overall faster approach towards success cues—a typical finding in AATs reflecting SR compatibility (Aubé et al., 2019; Krieglmeyer & Deutsch, 2010; Phaf et al., 2014; Rougier et al., 2018). Interestingly, prompt avoidance of failure cues was facilitated by higher levels of VN (*r* = −0.15) as well as the fear of shame or embarrassment (*r* = −0.18). This finding could indicate an increased vigilance towards failure cues, which vulnerable narcissists—who fear to embarrass themselves when making mistakes or displaying personal flaws—aim to avoid at all costs. Again, a different pattern of results emerged with respect to GN: higher expressions of GN were linked to faster overall responses in the AAT irrespective of the experimental condition. Therefore, our study provides no support for failure avoidance (or in turn an implicit attraction to success) in individuals with high trait levels of GN. The general velocity effect of GN could instead be related to extraversion, a core foundational personality trait of GN (Miller et al., 2011, 2021), which has been linked to faster movement times in SR compatibility tasks (Doucet & Stelmack, 1997, 2000). We note that studies employing AAT paradigms typically report difference scores, which capture the relative direction of behavioral tendencies and have been proposed as indirect measures of stimulus valence or affective interpretations of stimuli (Phaf et al., 2014). A limitation of this approach is that difference scores assume that approach and avoidance lie on opposite ends of a single continuum (Najmi et al., 2010), thereby failing to differentiate between distinct forms of avoidance. Since our focus was on a specific aspect of avoidance, we analyzed response latencies separately for each AAT condition. This procedure is consistent with prior work (e.g., Najmi et al., 2010; Struijs et al., 2017), but may reduce comparability with studies that report only difference scores.

### 4.1. Limitations

Although our sample size (*N* = 213) was adequate to test most of the a-priori hypotheses with satisfactory power, simulation research suggests that a sample of approximately 250 participants is needed to obtain stable correlation estimates in typical research contexts (Schönbrodt & Perugini, 2013). Our sample thus fell slightly below this benchmark. To account for this limitation, we applied bootstrapping to compute bivariate correlation coefficients (see Table 1). Importantly, the stability of correlation estimates also depends on the underlying effect size (Schönbrodt & Perugini, 2013). Given that the self-report correlations in our study were all in the medium-to-large range, the present sample size can be considered sufficient to detect robust effects. By contrast, the comparatively weaker associations between self-report and AAT data should be interpreted with caution. Moreover, pre-analysis estimations of sample size requirements indicated a larger *N* of at least 250 to detect a small-to-mediocre indirect effect of VN on socially prescribed perfectionism mediated by FIOLI (see Methods). Nonetheless, the mediation analyses revealed a significant mediation effect of FIOLI (see Figure 2), suggesting that the effect we found (*a* × *b* = 0.15) might have been even more substantial than expected. In contrast, the size of correlations detected between self-reported traits (e.g., GN and VN) and AAT response latencies we observed were relatively small, which however seems to be the rule rather than the exception when assessing relationships between cross-domain measures (e.g., behavioral measures and self-report data; Dang et al., 2020). Moreover, Hedge et al. (2018) suggested that experimental paradigms that usually produce large within-person effects, such as the SR-compatible AAT effects also found in the present study (see Supplemental Figure S1), are often less effective in detecting individual differences. Notably, this limitation generally applies to all the relationships of AAT response latencies we examined—with both GN and VN. Still, distinct AAT response patterns emerged, substantiating the support for distinct mental processes related to GN and VN.

Some methodological aspects of the current study limit the generalizability of our findings. First and foremost, although the hypotheses of this study were preregistered, the registration form provided limited information with regard to data preprocessing, sampling, and the analytic strategy, for which more details could have been provided a-priori. To foster open science practices, however, study materials, data, and analysis scripts were made publicly available. With regard to the sampling strategy and data collection mode, a major disadvantage of convenience sampling, as used in the present research, is the limited representativeness due to participants being selected based on availability and proximity. Like most studies on narcissism, in particular those linking it with perfectionism (Flett et al., 2014; Sherry et al., 2014; Smith et al., 2016; Vecchione et al., 2023), the current hypotheses were tested in a predominantly WEIRD (Western, educated, industrialized, rich, and democratic) sample, limiting cross-cultural generalizability of the present results and conclusions. Future studies should test the associations between different dimensions of narcissism, fear of failure, and perfectionism established here in small-scale and/or collectivist societies and in non-Western countries. Moreover, as the study was conducted online, data collection did not occur under completely standardized conditions. Even though all participants were instructed to complete both parts of the study, the survey and the AAT paradigm, alone, focused, and sealed off from any external disturbances, we had no means of controlling participants’ compliance with these terms. This constraint is not limited to, but is particularly relevant for the acquisition of sensitive reaction time data. Notably, a recent study (Sharpe et al., 2025) reported null-to-small differences in statistical results between self-report surveys conducted either remotely or in a typical laboratory setting. The authors reported, however, a slightly larger proportion of data flagged as “invalid” in studies with remote participation. Consequently, we employed different data quality checks, such as invariant responding analyses or the exclusion of response latency and accuracy outliers in the AAT. Finally, we acknowledge that narcissism was assessed using a single self-report measure, the PNI, which is considered to be biased toward capturing narcissistic vulnerability (Miller et al., 2017). Future research should incorporate instruments that more clearly distinguish between GN and VN and/or draw on more recent three-factor conceptualizations of narcissism (for an overview, see Miller et al., 2021).

### 4.2. Summary and Future Directions

In line with previous theoretical and empirical work (e.g., Cain et al., 2008; Miller & Campbell, 2008; Miller et al., 2011; Weiss & Miller, 2018), our study emphasizes the importance of discriminating between narcissistic grandiosity and narcissistic vulnerability. With respect to these two components of narcissism, fear of failure appears to be more relevant for VN than GN. Individuals high on VN are afraid of failure-triggered embarrassment and a loss of social significance. These anxious anticipations largely explain why vulnerable narcissists strive to appear perfect in front of others and aim to avoid failure at all costs. This suggests that vulnerable narcissists may employ perfectionism as an emotion regulation strategy (i.e., as a means of coping with their fear of failure). Given that socially prescribed perfectionism is considered the most maladaptive perfectionist style (Juwono et al., 2023; Stoeber, 2015) and is manifested in a variety of mental disorders, the present study clarifies some processes linking vulnerable narcissism to—in particular internalizing—psychopathology (Kaufman et al., 2020; Miller et al., 2018; Weiss & Miller, 2018). An interesting question arising from this conclusion is whether interventions tailored to modify these maladaptive regulation processes or dysfunctional cognitions are effective in buffering negative consequences of VN. Unlike vulnerable narcissists, grandiose narcissists tend to set high standards for others and do not appear to use perfectionism as a strategy to regulate failure-related fears. Since other-oriented perfectionism in GN cannot be explained by fear of failure, future studies could examine the factors motivating grandiose narcissists to impose high demands on others, a behavior that may strain social relationships and hinder personal development (Stoeber, 2015). Taken together, the findings of the present study add to the growing body of evidence on the differences between GN and VN (e.g., Cain et al., 2008; Miller et al., 2011, 2021). Future research should elucidate whether such differences warrant the assumption of distinct narcissism subtypes. Studies implementing person-centered approaches (for a recent example, see Maples et al., 2025) could aim to identify latent trait profiles of narcissism. Such research might shed light on the debate (for example, see Cain et al., 2008; Miller et al., 2021) on whether GN and VN should be considered different variants of narcissism or if they represent two sides of the same coin (i.e., different dimensions of the same trait that may occur within the same individual) and fluctuate over time. The present findings generally corroborate the notion that GN and VN are linked to distinct aspects of psychopathology (Kaufman et al., 2020).

## Figures and Tables

**Table 1:** Descriptive statistics and bivariate interrelations of study variables.

Item	*M*	*SD*	*α*	(1)	(2)	(3)	(4)	(5)	(6)	(7)	(8)	(9)	(10)	(11)	(12)	(13)
(1) PNI-GN	3.33	0.71	0.91	—												
(2) PNI-VN	3.05	0.89	0.94	**0.67**	—											
(3) OOP	3.15	0.59	0.78	**0.31**	**0.22**	—										
(4) SPP	2.79	0.75	0.88	**0.31**	**0.55**	**0.22**	—									
(5) SOP	3.75	0.84	0.90	**0.36**	**0.34**	**0.33**	**0.24**	—								
(6) FDSE	2.49	0.93	0.78	**0.35**	**0.61**	**0**.02	**0.48**	**0.18**	—							
(7) FIOLI	2.03	0.91	0.89	**0.43**	**0.62**	**0.22**	**0.68**	**0.18**	**0.52**	—						
(8) FUIO	2.22	0.94	0.86	**0.36**	**0.57**	***0.16***	**0.68**	**0.19**	**0.56**	**0.76**	—					
(9) FSE	2.78	1.00	0.88	**0.47**	**0.70**	***0.15***	**0.54**	**0.34**	**0.64**	**0.66**	**0.64**	—				
(10) Appr. Success	695	126	.	**−0.19**	−0.12	−0.00	−0.03	−0.02	−0.04	−0.03	−0.00	**−0.17**	—			
(11) Avd. Failure	744	139	.	***−0.17***	***−0.15***	0.09	−0.01	−0.01	−0.05	−0.01	0.03	**−0.18**	**0.88**	—		
(12) Appr. Failure	799	162	.	**−0.19**	−0.06	0.06	−0.04	−0.02	−0.02	−0.01	0.02	−0.11	**0.72**	**0.75**	—	
(13) Avd. Success	799	151	.	**−0.19**	−0.12	0.07	−0.00	−0.07	−0.03	−0.02	0.01	−0.14	**0.70**	**0.76**	**0.88**	—

Notes: (1) and (2)—Subscales of the Pathological Narcissism Inventory (PNI): grandiose (GN) and vulnerable narcissism (VN); (3)–(5)—dimensions of the Multidimensional Perfectionism Scale: other-oriented (OOP), socially prescribed (SPP), and self-oriented perfectionism (SOP); (6)–(9)—hypothesis-relevant dimensions of the Performance Failure Anxiety Inventory (PFAI): fear of devaluing one’s self-estimate (FDSE), fear of important others losing interest (FIOLI), fear of upsetting important others (FUIO), and fear of shame and embarrassment (FSE); (10)–(13)—median reaction times (in ms) from each AAT condition (Appr.—approach; Avd.—avoid). *α* indicates internal consistency (Cronbach’s *α* for self-report scales, Spearman–Brown coefficient for reaction times). Coefficients with *p* < 0.01 are displayed in bold and coefficients with *p* < 0.05 are displayed in bold and italic font (both two-sided). Bootstrapping with 1000 samples and bias-corrected and accelerated confidence intervals was used.

**Table 2:** Multiple regression analyses predicting perfectionism subtypes from grandiose and vulnerable narcissism.

Outcome Variable (Perfectionism Subtype)												
Predictor	*b*	OOP *^a^*		*p*	*b*	SPP *^b^*		*p*	*b*	SOP ^*c*^		*p*
		*SE*	*β*			*SE*	*β*			*SE*	*β*	
(1) Grandiose	0.25	0.07	0.30	<0.001	−0.12	0.08	−0.11	0.14	0.29	0.10	0.24	0.006
(2) Vulnerable	0.01	0.06	0.02	0.82	0.52	0.07	0.62	<0.001	0.16	0.08	0.17	0.047

Notes: *N* = 213. OOP—other-oriented perfectionism; SPP—socially prescribed perfectionism; SOP—self-oriented perfectionism. *^a^ R*^2^ = 0.10; *^b^ R*^2^ = 0.30; *^c^ R*^2^ = 0.14, all *p* < 0.001. Significance tests were two-sided. Vulnerable narcissism, perfectionism, and fear of failure.

## Data Availability

The preregistration document, analytic code, study materials, and original data presented in the study are openly available from the Open Science Framework (OSF) at https://osf.io/w2459/ (accessed on 1 September 2025).

## Notes

1 Please note that with regard to Hypothesis 1 (H1), only FIOLI and FUIO were included in the initial preregistration. Because some research indicates FSE as the most critical fear in the context of perfectionism (e.g., Sagar et al., 2010), we expanded H1 by FSE as a third supposed mediator.

2 Word frequency was determined for each word using a lexical corpus of the (Leipzig Corpora Collection, 2011). Hereby, frequency information is based on the word list of the largest corpus available in the German language.

3 The appropriateness of one-sided tests—even in the event of directional hypotheses—is a matter of debate (e.g., Greenland et al., 2016; Ruxton & Neuhäuser, 2010). Some researchers recommend defaulting to two-sided tests as a more conservative way of hypothesis testing that preserves the ability to detect unexpected results (e.g., see Ruxton & Neuhäuser, 2010).

4 Differences in sample sizes required for the different mediation models result from differences in effect sizes, i.e., the size of bivariate correlations between predictor (GN, VN), outcome (types of perfectionism), and the respective mediator considered in the model. Estimates for these bivariate correlations were extracted from the literature (Conroy et al., 2007; Henschel & Iffland, 2021; Smith et al., 2016). A screenshot with the relevant input parameters used in these power analyses is provided at https://osf.io/w2459/?view_only=4f728d97bed94a2db4c7d5f900391fde (accessed on 1 September 2025). The shiny app was accessed through https://schoemanna.shinyapps.io/mc_power_med/ (accessed on 1 September 2025).

## Supplementary Materials

The following supporting information can be downloaded at https://www.mdpi.com/article/10.3390/bs15091214/s1. Figure S1: Average response times (RTs) in the approach-avoidance task (AAT) by experimental condition.
