# Supplementary material for: Am I (Not) Perfect? Fear of Failure Mediates the Link Between Vulnerable Narcissism and Perfectionism
Source: Behav Sci (Basel). 2025 Sep 6;15(9):1214. doi: 10.3390/bs15091214 (PMC12466693; doi:10.3390/bs15091214)
Supplement: Supplementary file 1 [file behavsci-15-01214-s001.zip › behavsci-3741549-supplementary.pdf]

**Supplemental Material**

**Figure S1**

*Average Response Latencies in the Approach-Avoidance Task (AAT) by Experimental Condition*

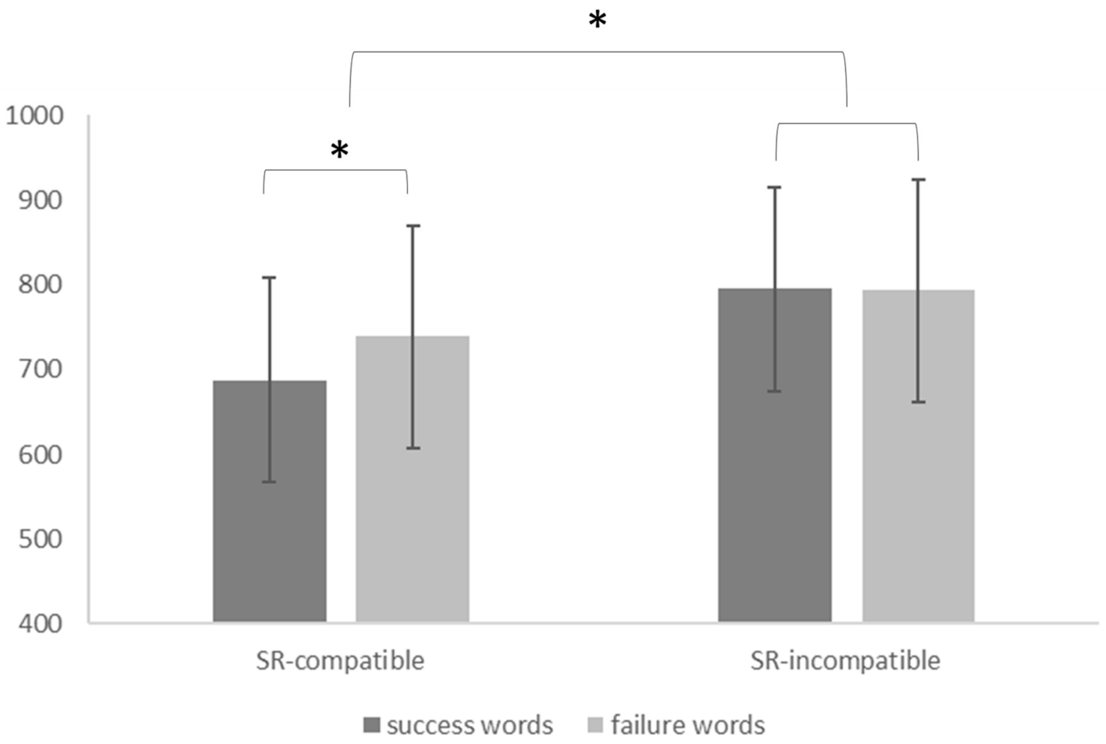


*Note*. SR-compatible – AAT block in which participants were asked to approach success words and avoid failure words; SR-incompatible – AAT block in which participants were asked to approach failure words and avoid success words. Response latencies are shown in milliseconds. Error bars indicate standard deviations.
